# Impact Assessment of vB_KpnP_K1-ULIP33 Bacteriophage on the Human Gut Microbiota Using a Dynamic In Vitro Model

**DOI:** 10.3390/v15030719

**Published:** 2023-03-10

**Authors:** Fanny Laforêt, Céline Antoine, Sarah Lebrun, Irma Gonza, Elizabeth Goya-Jorge, Caroline Douny, Jean-Noël Duprez, Marie-Louise Scippo, Bernard Taminiau, Georges Daube, Abdoulaye Fall, Damien Thiry, Véronique Delcenserie

**Affiliations:** 1 Laboratory of Bacteriology, Department of Infectious and Parasitic Diseases, FARAH, Faculty of Veterinary Medicine, University of Liège, 4000 Liège, Belgium; 2 Laboratory of Food Quality Management, Department of Food Sciences, FARAH, Faculty of Veterinary Medicine, University of Liège, 4000 Liège, Belgium; 3 Laboratory of Food Analysis, Department of Food Sciences, FARAH, Faculty of Veterinary Medicine, University of Liège, 4000 Liège, Belgium; 4 Laboratory of Microbiology, Department of Food Sciences, FARAH, Faculty of Veterinary Medicine, University of Liège, 4000 Liège, Belgium; 5 FoodChain ID GENOMICS SA, En Hayeneux 62, 4040 Herstal, Belgium

**Keywords:** SHIME^®^, bacteriophage, hypervirulent K1 *Klebsiella pneumoniae*, intestinal microbiota, α-diversity, β-diversity, short-chain fatty acids

## Abstract

New control methods are needed to counter antimicrobial resistances and the use of bacteriophages as an alternative treatment seems promising. To that end, the effect of the phage vB_KpnP_K1-ULIP33, whose host is the hypervirulent *Klebsiella pneumoniae* SA12 (ST23 and capsular type K1), was assessed on intestinal microbiota, using an in vitro model: the SHIME^®^ system (Simulator of the Human Intestinal Microbial Ecosystem). After stabilization of the system, the phage was inoculated for 7 days and its persistence in the different colons was studied until its disappearance from the system. The concentration of short chain fatty acids in the colons showed good colonization of the bioreactors by the microbiota and no significant effect related to the phage treatment. Diversity (α and β), the relative abundance of bacteria, and qPCR analysis targeting different genera of interest showed no significant variation following phage administration. Even if further in vitro studies are needed to assess the efficacy of this phage against its bacterial host within the human intestinal ecosystem, the phage ULIP33 exerted no significant change on the global colonic microbiota.

## 1. Introduction

Even if antibiotic resistance is a natural adaptation of the bacterium, their overuse accelerated the process, thus new therapies are needed. In this context, phage therapy seems promising [1,2].

In the case of per os treatments with bacteriophages, these are directly exposed to the intestinal microbiota. Thence, even if lytic phages are well-known to be specific to their host and to be part of the intestinal microbiota [3], the use of phages targeting enterobacteria, a commensal bacterial family of the human gastro-intestinal tract [4], should be performed with caution. Currently, only a few works have studied the impact of bacteriophages on the intestinal microbiota. When one of them do not exert any impact [5], others, on gnotobiotic mice, bring out community changes in the microbiota, not only on target bacteria but also on others present in the microbiota [6,7,8]. This can be an issue if the new targeted bacterium is a beneficial bacterium for host health and if these changes disrupt the normobiosis of the gut microbiota.

To assess the efficacy of new phages, and their effect on human intestinal microbiota, several in vitro, in vivo, or ex vivo models exist. In vivo and ex vivo models (using humans, animals or organs biopsy) are limited mainly because of ethical restrictions. Regarding in vitro colon fermentation models, 2 types of systems exist: the static and the dynamic models. The first, also called batch fermentation models, such as CoMiniGut [9], are mainly used for preliminary investigations, to simulate the fermentations usually observed in the distal part of the colon for a short period of time and using a consensus protocol (InfoGest) [10,11,12]. In contrast, dynamic models seek to mimic continuous fermentations by a constant addition of nutrients to the system [12]. From mini fermentation models such as Mini Bio with 32 bioreactors [13,14] or MiPro using 96-deep well plates [15] to bigger models similar to the mono-compartmental colonic ARCOL (Artificial Colon) [16,17] or the 4 fermenters TIM-2 (TNO intestinal model 2) [16,17,18] or the upper colonic PolyFermS [19], other more complex models exist. These complex models simulate the entire digestive system including the digestion and the fermentation processes such as the SIMulator of the Gastro-Intestinal tract (SIMGI) (often used for its in vitro stomach properties) [20] or the SHIME^®^ (Simulator of Human Intestinal Microbial Ecosystem).

The SHIME^®^ model is a computer-controlled system that allows the simulation of the stomach, ileum, ascending colon, transverse colon, and descending colon [21]. Mucin beads can be added to the system (M-SHIME^®^) to maintain the mucin-associated ecosystem. Sometimes, dialysis membranes are also added to simulate the passive absorption of the metabolites. This model is, therefore, one of the most complete models to study the microbiota and is validated to study the impact of various treatments on the microbiota [11,22]. Microbiota profiling, α and β-diversities, semi-quantification of specific genera using qPCR, phage titrations, and short-chain fatty acid production can be deeply analyzed from SHIME^®^ samples.

Microbiota profiling (relative abundance of the different bacteria) and diversity parameters are obtained using 16S RNA gene sequencing. These are well-known tools to study the microbiota of different bacterial ecosystems such as food samples [23], skin [24], or intestinal microbiomes [25].

Semi-quantitative methods such as qPCR are used to follow specific genera involved, for example, in gut health [26,27,28,29,30,31] or diseases [30,31,32].

Short-chain fatty acids (SCFA) such as acetate, propionate, and butyrate are the main end products of the degradation of polysaccharides by the bacteria of the microbiota through fermentation and play important roles on hosts’ health [33,34]; hormones regulation (for example leptin or insulin), anti-inflammatory properties (through the inhibition of the histone deacetylase), antimicrobial peptides or intestinal mucus productions, energy source for the colonocytes or tight junction activation.

The SHIME^®^ model has previously been used to study phage therapy in intestinal conditions. Verthé et al., studied the persistence and impact of a phage targeting a strain of *Klebsiella aerogenes* (previously named *Enterobacter aerogenes*) after a single injection into the model [35]. A second study assessed the interaction of a phage cocktail with *Salmonella* Typhimurium and the microbiota [36]. Then, Federici et al. [37] studied the activity of 2 phages against *Klebsiella pneumoniae* after their passage through the proximal or distal colonic microbiota.

The main aim of this work was to assess the impact and the persistence of the phage vB_KpnP_K1-ULIP33 in the gut microbiota after daily injections for a period of 7 days, using the SHIME^®^ model. This phage was isolated from sewage water using a hypervirulent *K. pneumoniae* ST23 (SA12, SB4385) [38] and showed the capacity to increase the survival of *Galleria mellonella* larvae infected with this capsular type K1 strain [38]. Potential changes in the microbiota biocenosis due to the phage treatment were investigated using microbiota profiling, phage titrations, SCFA analysis and semi-quantification of specific genera by qPCR. In addition, the secondary goal of this study was to assess the repeatability of the model in the case of phage addition experiment using a technical triplicate.

## 2. Materials and Methods

### 2.1. Set-Up of the SHIME^®^ Model (Simulator of Human Intestinal Microbiota Ecosystem)

**Figure 1:**
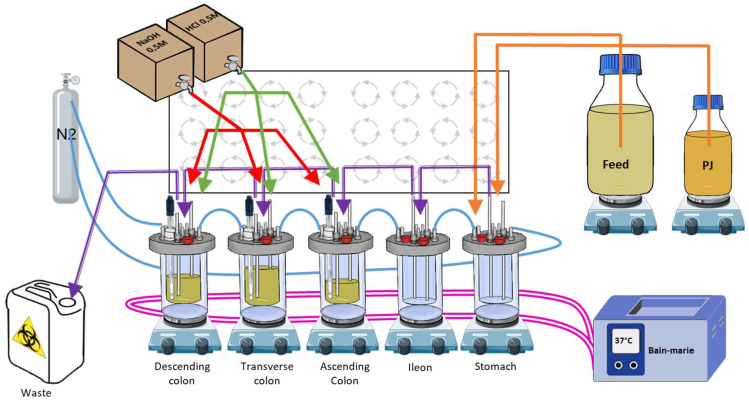
Schematic representation of a human SHIME^®^ system. The 5 bioreactors are represented on their magnetic stirrers: the 3 colons, the stomach, and the ileum. Pink lines: warm bath connections to maintain the temperature; blue lines: N_2_ flush connections for anaerobic conditions; purple lines: connections between the different bioreactors for transfers (till the waste); orange lines: connections to feed and pancreatic juice media for digestion simulation; green lines: connections for acidic and basic solution distribution to maintain the pH ranges. PJ: pancreatic juice.

The SHIME^®^ system was set up to mimic an adult gastrointestinal tract (Figure 1) and simulate the different conditions found in the stomach/duodenum (shortened as stomach), jejunum/ileum (shortened as ileum), and the 3 colons (ascending (AC), transverse (TC) and descending (DC)) as described previously [21,22]. The experiment was performed in triplicate.

In brief, 5 bioreactors, each representing a part of the digestive tract, were airtightly sealed, continuously stirred using magnetic stirrers, maintained at 37 °C, connected to each other, and maintained in anaerobic conditions using a daily flow of nitrogen. The stomach bioreactor was connected to two different media. The first, called feed (Prodigest, Ghent, Belgium; ref PD-NM001B), was discharged into the system 3 times a day to mimic food intake. The second, a synthetic pancreatic juice, called PJ, a mix of NaHCO_3_-pancreatin-bile salts (Prodigest, Ghent, Belgium), was discharged into the system 3 times a day to mimic digestion. The colon bioreactors were inoculated with human feces and the pH of each colon was maintained in a specific range, close to in vivo conditions: AC: 5.6–5.9, TC: 6.15–6.4, and DC: 6.6–6.9.

### 2.2. Feces Collection

A fresh inoculum was obtained from a healthy 34-year-old volunteer donor (female) with no history of antibiotic use for at least 6 months. This collection of feces and its use in the model was approved by the ethical committee of the University of Liège (ULiège, Liège, Belgium; file number 2022/274). Directly after the sampling, the feces were stored in anaerobic jars and sent to the laboratory for immediate processing. The inoculum was diluted to 20% (*w/v*) in phosphate buffer and then homogenized in a stomacher (VWR, Leicestershire, UK) for 10 min. The fecal suspension was then macro-filtered at 250 μm (VWR, Leicestershire, UK). The supernatant was collected and placed in glycerol (15% (*v*/*v*)) to be frozen (25 mL, 40 mL, and 30 mL of supernatant were necessary for the inoculation of the AC, TC, and DC, respectively). On the inoculation day, the defrosted fecal suspension was inoculated in the different colon bioreactors already filled with automatic pH adjusted culture media. Following inoculation, the systems were maintained for a period of 23 days to allow the stabilization of the introduced fecal community.

### 2.3. Bacteriophage Characteristics and Inoculation

The bacteriophage studied in this experiment was vB_KpnP_K1-ULIP33 [38]. This phage was isolated in sewage from Rueil-Malmaison (France) using the bacterial strain *Klebsiella pneumoniae* SA12 (SB4385), a capsular type K1 ST23 bacterium. The phage was amplified using a classical amplification process: overnight contact of the phage with its host bacteria (SB4385) and then filtration of the solution using 0.22 µm microfilters. This amplified lysate was semi-purified with a 30% sucrose cushion using an ultracentrifugation cycle (50,000× *g* for 4 h). The obtained pellet was then washed through another ultracentrifugation cycle (4 h at 50,000× *g*) in phosphate-buffered solution (PBS). Next, the phage was diluted to the desired concentration using PBS.

**Figure 2:**
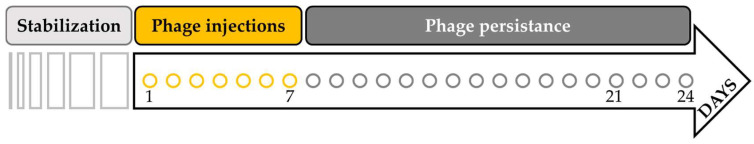
Timeline of the SHIME experiment. After the inoculation of human feces in the ascending, transverse, and descending colons, the microbiota needed 23 days for stabilization. Then, 10 mL at 10^9^ (PFU/mL) of the phage ULIP33 was injected in the ascending colon for 7 days (from day 1 to day 7). The experiment was maintained until the disappearance of the phage from the system (corresponding to day 24 for repetition 1 and day 21 for repetition 2 and 3).

After inoculation of the human feces in the ascending, transverse, and descending colons, 23 days of microbiota stabilization were necessary (Figure 2). Next, 10 mL of the phage were inoculated at 10^9^ (PFU/mL) once a day, for 7 days (from day 1 till day 7), in the ascending colon, just before the transfer of the feed media in the AC. The persistence of the phage was then studied until its disappearance (until day 24 for repetition 1 and day 21 for repetition 2 and 3). To that end, titrations, on SA12 bacterial overlays, were carried out: on day 1 (4 h after the first injection), at days 2, 4, and 6 during the injection week, at day 8, and from day 11 until the end of the experiment.

Based on Verthé et al. [35], a mathematical model was applied to compare the theoretical elimination of the phage by the transit when considered as an inert molecule. The theoretical persistence (concentration in PFU/mL) of the phage (meal after meal of 200 mL) in the different bioreactors was mathematically translated as follows:

$$[\mathrm{AC}]=\frac{\left[{\mathrm{AC}}^{\prime }\right]\;\times 500-[{\mathrm{AC}}^{\prime }]\times 200}{500}$$

$$[\mathrm{TC}]=\frac{\left[{\mathrm{TC}}^{\prime }\right]\;\times 800+[{\mathrm{AC}}^{\prime }]\times 200-[{\mathrm{TC}}^{\prime }]\times 200}{800}$$

$$[\mathrm{DC}]=\frac{\left[{\mathrm{DC}}^{\prime }\right]\times 600+[{\mathrm{TC}}^{\prime }]\times 200-[{\mathrm{DC}}^{\prime }]\times 200\;}{600}$$

With [AC], [TC], and [DC] being the concentrations (in PFU/mL) during the meal for AC, TC or DC colons; [AC′], [TC′], and [DC′] being the concentrations (in PFU/mL) during the previous meal; and 500, 600 and, 800 being the contents (in mL) in the bioreactors for AC, TC, and DC colon, respectively. The volume of 200 mL is the volume transferred from one bioreactor to another (140 mL of feed and 60 mL of PJ).

An additional mathematical model was formulated to calculate the expected concentration of the phage in the total volume of 1900 mL at the end of the 7 days of phage inoculation. This model considered the dilutions of the phage due to the 3 meals, the loss toward the waste material, and the gain through the injections:

$$PI=\left[\mathrm{LM}\right]\times 1900+10^{10}\;$$

$$Loss=200\times \frac{PI}{1900}$$

$$\left[\mathrm{AM}\right]=\frac{PI-Loss}{1900}$$

With “*PI*” being the PFU post-injection in the bioreactors; [LM] the concentration after the last meal; “*Loss*” being the PFU lost toward the waste material; and [AM] the final concentration after the meal. 

### 2.4. Short-Chain Fatty Acid Analysis

Samples from the colon bioreactors of the SHIME^®^ were collected 3 times per week and were analyzed for their SCFA content. The studied short-chain fatty acids (SCFA) were acetic, propionic, and butyric acid. Solid phase micro-extraction (SPME) fiber (Thermo Scientific, Merelbeke, Belgium) was used to extract the components and the Focus CG gas chromatograph (GC) (Thermo Scientific, Merelbeke, Belgium) with a Supelcowax-10 column (Thermo Scientific, Merelbeke, Belgium) was used to separate them. Finally, the ion trap PolarisQ mass spectrometer (MS) (Thermo Scientific, Merelbeke, Belgium) allowed their analysis [39]. To prepare the samples, 25 µL of SHIME^®^ sample, 40 µL of 2-methylvaleric acid (0.2 mg/mL) as internal standard, 15 µL of H_2_SO_4_ at 0.9 M and 920 µL of water were pipetted together into a 20 mL glass vial. The mixture was vortexed and then placed in the SPME-CG-MS for analysis. The lower limits of quantification (LLOQ) were 2, 1, and 1 mM for acetate, propionate, and butyrate, respectively, and the upper limits of quantification (ULOQ) were 120, 55, and 40 mM.

### 2.5. 16S rRNA Gene Sequencing

Two mL of samples collected from the colon bioreactors were pelleted and analyzed for their microbiological content. The endpoints chosen for the microbiota profiling were day 1 as control, day 8, day 15, and day 24 or 21 (last day of the experiment).

The DNA was extracted using the PSP spin Stool DNA Basic kit associated with the Stool DNA Stabilizer (Invitek Molecular, Berlin, Germany). After purification, the quality and the quantity of DNA were measured using the Nanodrop 2000 (Thermo Scientific, Merelbeke, Belgium). The V1–V3 hyper variable region of the 16S rDNA bacterial gene were amplified using the E9–E29 and E514-530 primers [40] and amplification products (amplicons) purified with a Wizard SV PCR purification kit (Promega, Alken, Belgium). After this step, the amplicons were submitted to a second PCR for indexing, using the Nextera XT index kit with dual 8-base indices (Illumina, San Diego, CA, USA). After cleaning with AMPure XP beads (Beckman Coulter, Indianapolis, IN, USA), the librairies were quantified, pooled and sequenced by 2 × 300 bp paired end sequencing on the MiSeq platform using MiSeq v3 Reagent Kit (Illumina, San Diego, CA, USA). Using Mothur package v 1.47, the sequences were trimmed, filtered, and cleaned for chimeras (https://www.mothur.org, accessed on 30 June 2021). The final reads were clustered into operational taxonomic units (OTUs) at a 0.01 distance unit cutoff. Using BLASTN algorithm, a representative sequence for each OUT was compared to SILVA dataset (SILVA v138) [41]. Each OTU was analyzed as a proportion of reads to deduce the relative abundance.

### 2.6. qPCR of Selected Taxa

The qPCR were performed using the same DNA extracts as those used for 16S rRNA gene sequencing. The assays were performed in 96 well plates (Nippon Genetics Europe, Düren, Germany) in technical duplicates of the triplicate SHIME samples (*n* = 6). The wells were filled with 10 µL of Takyon™ No ROX SYBR MasterMix (Eurogentec S.A., Seraing, Belgium), reverse and forward primers of each selected sequence (from 0.3–0.5 µM) (Eurogentec S.A., Seraing, Belgium), 2.5 µL of the DNA sample (at 4 ng/µL) and diluted in molecular biology grade water to reach 20 µL per well. In all assays, a negative control was included with molecular biology grade water instead of the DNA product.

The qPCR protocol included an initial denaturation step at 95 °C for 5 min, followed by 35 cycles of: denaturation at 95 °C for 15 s, annealing at optimal primer temperature for each targeting species (Table S1) for 15 s, elongation at 72 °C for 30 s followed by a final elongation step at 72 °C for 5 min [42,43]. An analysis of the melt curve (ranging from 65 °C to 95 °C) was made to evaluate the specificity of the amplified products.

The 2^−ΔΔCt^ method [44] was used to calculate the relative changes in the target populations normalized to total bacteria population after phage injections.

Eleven taxa were followed by qPCR: *Akkermansia muciniphila* [45], *Bifidobacterium* [46], *Faecalibacterium prausnitzii* [47] and *Oscillospira* [48] as gut health biomarkers; *Bacteroides/Prevotella* [47], *Phascolarctobacterium faecium* [49] and *Veillonella* [50] as SCFA producer biomarkers (especially propionate production); *Ruminococcus* [51] and *Mucispirillum schaedleri* [52] as chronic inflammation biomarkers; and *Escherichia/Shigella* [47] as acute inflammation biomarker and *Klebsiella pneumoniae* complex [53] to test the specificity of ULIP33 phage.

### 2.7. Statistical Analysis

Statistical analyses were performed using R with “vegan” and “Rcmdr” packages [54]. A significant threshold of 0.05 was applied for all the statistical tests and the Bonferroni correction was applied if needed. The graphical representations of the SCFA were performed using GraphPad Prism version 8.0.2 for Windows, GraphPad Software (San Diego, CA, USA).

Regarding the metagenetic results, the β-diversity and the α-diversity were studied. The β-diversity (based on microbial diversity between the samples) was visualized using a “Non-metric Multidimensional Scaling” (NMDS). Then, the “Analysis of the MOlecular VAriance” (AMOVA) was calculated based on the Bray–Curtis dissimilarly matrix and the homogeneity of the groups were then tested with the “HOmogeneity of the MOlecular VAriance” (HOMOVA) and the multiple comparison with Tukey–Kramer method when needed. For α-diversity, the Shannon diversity index, the Piélou index, the Simpson index, and the chao1 estimator were studied [55,56].

SCFA concentrations, relative quantification by qPCR and α-diversity index were analyzed to highlight changes due to the phage addition in each colon bioreactor. The first step was to investigate the normality of the residues of each distribution using a histogram, a quantile-quantile plot (QQ-plot), a boxplot, a Shapiro–Wilk test, and the homoscedasticity of the distributions. If the residues were normally distributed, a repeated-measures ANOVA was performed with paired Student’s *t*-test. If not normally distributed, a Friedman test and a Pairwise Wilcoxon Rank Sum Test were performed.

## 3. Results

### 3.1. Bacteriophage Titrations

**Figure 3:**
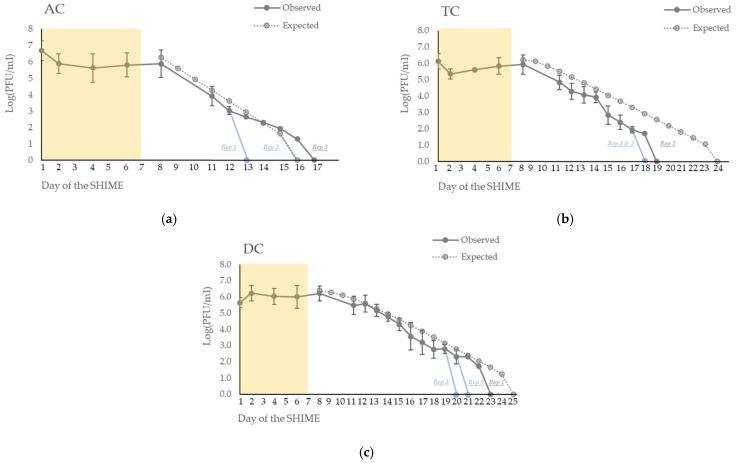
Persistence of the phage ULIP33 in ascending (AC) (**a**), transverse (TC) (**b**) and descending (DC) (**c**) colons in the SHIME^®^ model after one daily phage injection (10 mL at 10^9^ PFU/mL) in the ascending colon for 7 days. The mean concentrations (±SD) are presented in log (PFU/mL) for the observed data of the phage titrations (dark gray curves). The expected phage titrations (based on the mathematical model; dotted gray curve) are calculated based on the observed titrations of the phage at day 8. The yellow frame represents the phage treatment week; blue curves represent the fastest disappearance in the different repetitions.

During the week of injections, the concentration of the phage remained stable in all the fermenters (Figure 3) with an observed average concentration of 6.6 × 10^5^ PFU/mL in the total volume of the bioreactors (1.9 L). The theoretical expected average concentration during the week of injections was 9 × 10^6^ PFU/mL with an expected final concentration of 1.2 × 10^7^ PFU/mL on day 8.

After the end of the 7 days of inoculation, the phage gradually disappeared from the system in each colon (Figure 3). It disappeared at a faster rate than the mathematical expectation.

In the ascending colon (AC) (Figure 3a), the phages disappeared at day 13 and day 17 for, respectively, the first and the third repetition. In the second repetition, it disappeared at day 16, as expected through the mathematical model.

In the transverse colon (TC) (Figure 3b), the phages disappeared at day 18 in the first and third repetitions and at day 19 for the second repetition. In contrast, the expected wash-out calculated by the mathematical model was at day 24.

In the descending colon (DC) (Figure 3c), the phages disappeared at day 20 for repetition 3, at day 21 for repetition 2, at day 23 for repetition 1, and at day 25 for the expected wash-out obtained through the mathematical model.

### 3.2. Short-Chain Fatty Acid Profile

**Figure 4:**
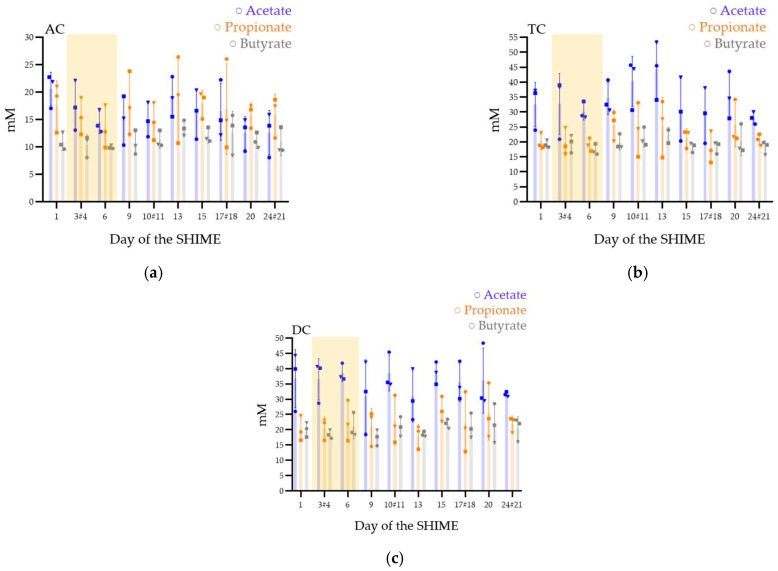
Short-chain fatty acid production (acetate, propionate, and butyrate) in the ascending (AC) (**a**), transverse (TC) (**b**), and descending (DC) (**c**) colons in the SHIME^®^ model after one daily ULIP33 phage injection (10 mL at 10^9^ PFU/mL) in the ascending colon for 7 days. Mean concentrations (±SD) are presented in mM for acetate (blue bars), propionate (orange bars), and butyrate (gray bars); all are quantified using SPME-GC-MS, 3 times a week. Round marks: data of the repetition 1; square marks: data of the repetition 2; triangle marks: data of the repetition 3; yellow frame represents the phage treatment week and # are the samples with a different sample date in the different triplicates.

In AC, the concentrations of the three mains different SCFA (acetate, propionate, and butyrate) were stable over time (Figure 4a). The means of the concentrations (in mM) at key day-point samples varying from 13 ± 4 to 21 ± 3 for acetate, 16 ± 4 to 18 ± 6 for propionate, and 11 ± 2 to 12 ± 1 for butyrate (Table 1).

In TC, after stopping the phage inoculation, the microbiota increased its production of acetate (Figure 4b). However, the means concentrations (in mM) varied from 28 ± 2 to 35 ± 5 for acetate, 20 ± 3 to 26 ± 5 for propionate, and 18 ± 2 to 20 ± 3 for butyrate (Table 1). 

DC (Figure 4c) was stable with means concentrations (in mM) between 31 ± 12 and 37 ± 10 for acetate, 20 ± 4 and 27 ± 4 for propionate, and 17 ± 3 and 22 ± 2 for butyrate (Table 1).

In the AC colons of the three repetitions a higher ratio of propionate to acetate was observed contrarily to the TC and DC (Table 1).

The performed statistical analyses, either ANOVA tests (for parametric distributions) or Friedman tests (for non-parametric distributions), did not reveal any significant changes in colons following phage treatments, for they all tested SCFA. Friedman tests were calculated on acetate and propionate production in the TC and butyrate production in DC. ANOVA tests were performed for all the other distributions.

### 3.3. Microbiota Ecosystem Analyses

#### 3.3.1. Microbiota Composition in the SHIME^®^ Model

Regarding the composition of the fecal inoculum from the donor, *Faecalibacterium* presented the higher relative abundance, followed by the *Bacteroides, Lachnospiraceae_ge, Agathobacter, Oscillospirales_ge, Subdoligranulum, Lachnoclostridium, Roseburia, Parabacteroides*, and *Lachnospiraceae_NK4A136_groups* (Figure S1). A total of 83 OTU was observed for this donor.

**Figure 5:**
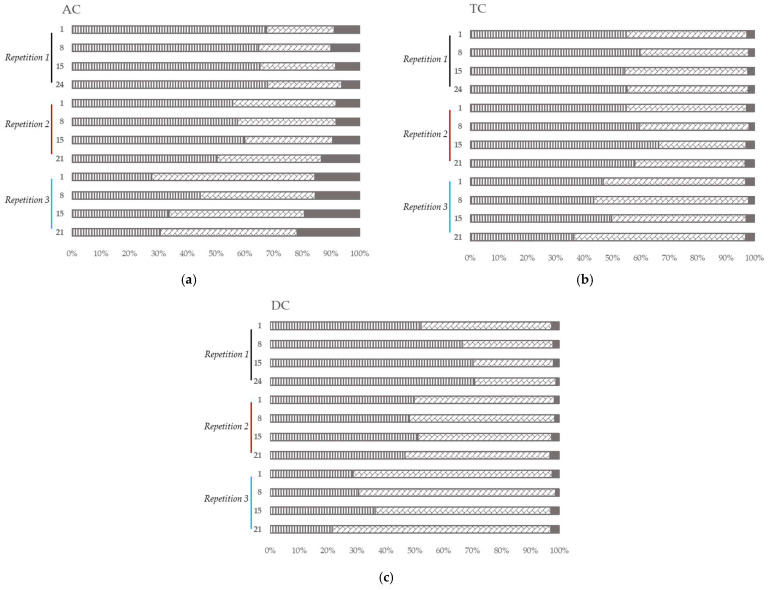
Microbial community analysis in ascending (AC) (**a**), transverse (TC) (**b**) and descending (DC) (**c**) colons assessed by 16S RNA Illumina Sequencing after one daily ULIP33 phage injection (10 mL at 10^9^ PFU/mL) in the ascending colon for 7 days. Relative abundance histogram at phylum level (in %). Black line: repetition 1; burgundy line: repetition 2 and light blue line: repetition 3. Vertical stripes marks: Firmicutes phylum; diagonal bricks marks: Bacteroidota phylum; plain gray color: Proteobacteria phylum; plain black color: other phyla.

Regarding the composition of the microbiota over time, at the phylum level, the Firmicutes and the Bacteroidota presented the highest relative abundance in all the colons for all the repetitions (Figure 5). The relative abundance of Firmicutes was higher than Bacteroidota, except for in the third repetition. The other phyla abundances were low except for the Proteobacteria in AC (Figure 5a).

In the first repetition, the relative abundance of the Firmicutes and the Bacteroidota remained stable in AC (Figure 5a) and TC (Figure 5b) over time. In DC (Figure 5c), the Firmicutes increased over time when the Bacteroidota decreased.

In the second and the third repetitions, the relative abundance of the Firmicutes and Bacteroidota stayed stable over time in all the colons (Figure 5). Nevertheless, in AC (Figure 5a), the relative abundance in Proteobacteria increased over time.

**Figure 6:**
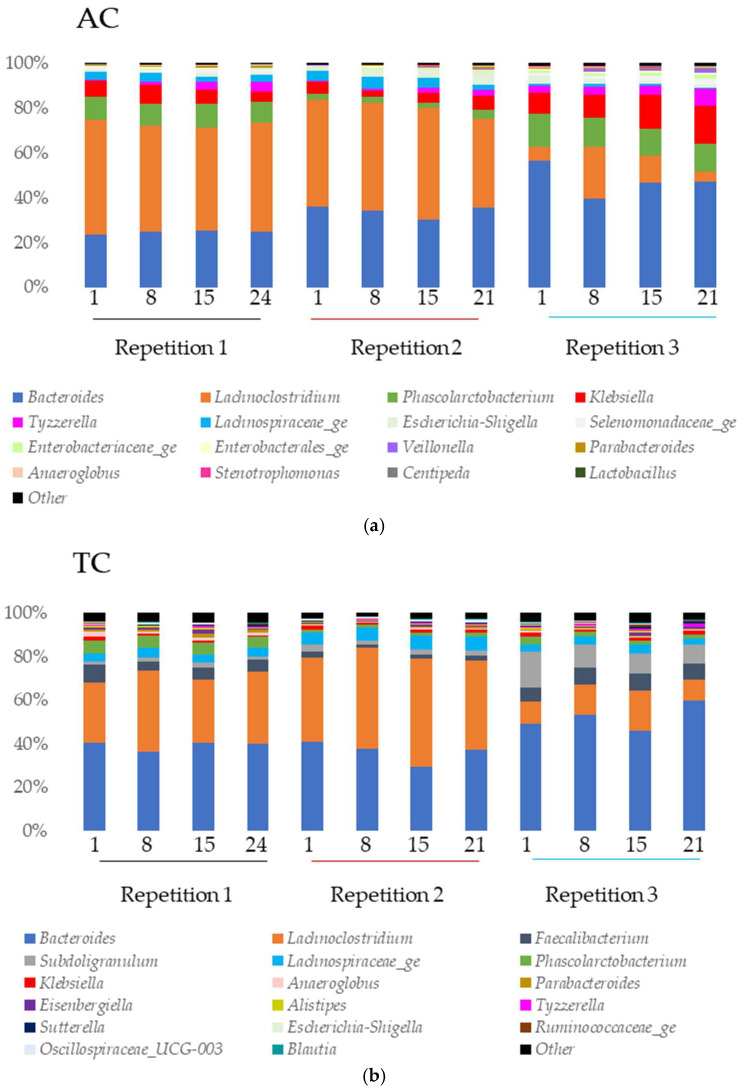

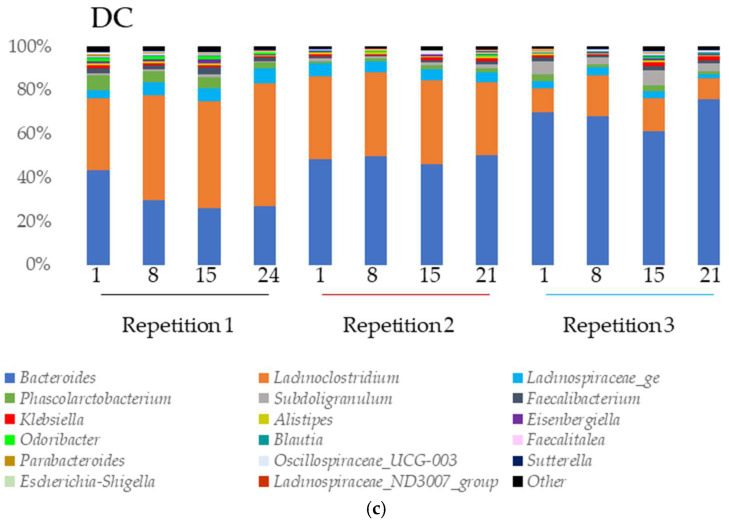
Microbial community analysis in ascending (AC) (**a**), transverse (TC) (**b**) and descending (DC) (**c**) colons assessed by 16S rRNA Illumina Sequencing after one daily ULIP33 phage injection (10 mL at 10^9^ PFU/mL) in the ascending colon for 7 days. Relative abundance (in %) histogram at genus level. Black line: repetition 1; burgundy line: repetition 2 and light blue line: repetition 3.

In all colons, at genera level (Figure 6), *Bacteroides* and *Lachnoclostridium* covered more than 70% of the relative abundance in the first and second repetitions. In the third repetition, *Lachnoclostridium* was found in a lower proportion in all the colons (compared to the other repetitions) while *Bacteroides* showed a higher proportion mainly in DC.

In AC (Figure 6a), *Klebsiella* and *Phascolarctobacterium* were found in high proportion in all the samples of the experiment. The abundance of *Lachnospiraceae_ge* was important in the first and second repetition. *Tyzzerella* was abundant in the first and third repetition while *Escherichia-Shigella* was abundant in the second and third.

In TC (Figure 6b), *Subdoligranulum* was found in high proportion in the second and the third repetitions. *Faecalibacterium* and *Lachnospiraceae_ge* were abundant in all the samples of all repetitions and *Phascolarctobacterium* in the first repetition.

In DC (Figure 6c), the main genus found were *Phascolarctobacterium* (all repetitions), *Lachnospiraceae_ge* (all repetitions), *Faecalibacterium* (repetition 1), and *Subdoligranulum* (repetitions 2 and 3).

#### 3.3.2. α-Diversity

The results of α-diversity index and OTU numbers at key-point samples are summarized in Table 2.

For the Shannon diversity index, the expected maxima predictions were 4, 4.6, and 4.9 for the AC, the TC, and the DC, respectively (with the maximum corresponding to ln(S), with *S* being the total number of species present in the bioreactor). In AC, the Shannon index varied between 1.44 ± 0.12 and 1.64 ± 0.11 while it varied between 1.67 ± 0.23 and 1.85 ± 0.20 in TC. Finally, DC the range was from 1.43 ± 0.17 until 1.68 ± 0.17.

The Piélou equitability index varied between 0.39 ± 0.03 and 0.45 ± 0.06, 0.32 ± 0,04 and 0.38 ± 0.05, and 0.40 ± 0.05 and 0.43 ± 0.04 for, respectively, the AC, TC, and DC.

Regarding the Simpson index, the obtained samples showed a variability from 0.65 ± 0.02 to 0.71 ± 0.02, from 0.68 ± 0.05 to 0.71 ± 0.04, and from 0.57 ± 0.12 to 0.66 ± 0.04 in AC, TC, and DC, respectively.

Finally, the chao1 estimator had a range between 31 ± 5.83 and 42 ± 4.16 in AC, 76 ± 3.91 and 82 ± 4.77 in TC, and 80 ± 11.15 and 113 ± 38.07 in DC.

For the inoculum, the values of the index were 2.58, 0.58, and 0.82 for the Shannon, Piélou, and Simpson index respectively, and 97 for the chao1 estimator. The number of OTU was 83.

Statistical analysis was applied to highlight diversity index changes following treatment. Depending on the normality and homoscedasticity of the distributions, either ANOVA tests, for the Piélou index and Chao1 estimator in the TC, or Friedman tests, for all other distributions, were applied. Independently of the statistical test applied, no significant changes in the different index were observed in all the colons and for all the selected bacteria.

#### 3.3.3. β-Diversity

**Figure 7:**
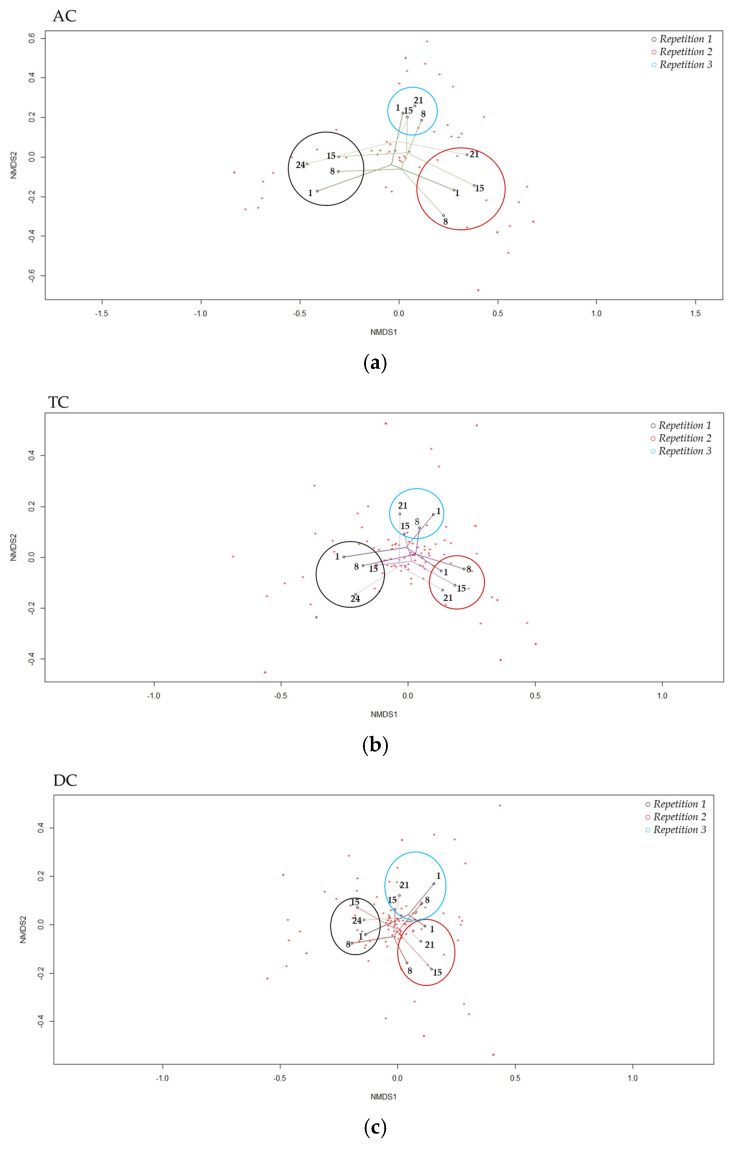
“Non-Metric Multidimensional Scaling”, NMDS, of β-diversity the in ascending (**a**), transverse (**b**) and descending (**c**) colons assessed by 16S rRNA Illumina Sequencing after one daily ULIP33 phage injection (10 mL at 10^9^ PFU/mL) in the ascending colon for 7 days. Black circle: repetition 1; burgundy circle: repetition 2 and light blue circle: repetition 3.

The Non-Metric multidimensional scaling, representing the β-diversity, showed that the bacterial diversity between the samples was close, regardless of the sampling day or repetition (Figure 7). In addition, the samples seemed to be strongly clustered by repetition. The groups clustering test (AMOVA) and the homogeneity test (HOMOVA) showed no significant results, even for the AC, TC, or DC (no significant genetic difference between the samples).

### 3.4. Evolution of Target Bacteria Followed by qPCR

**Figure 8:**
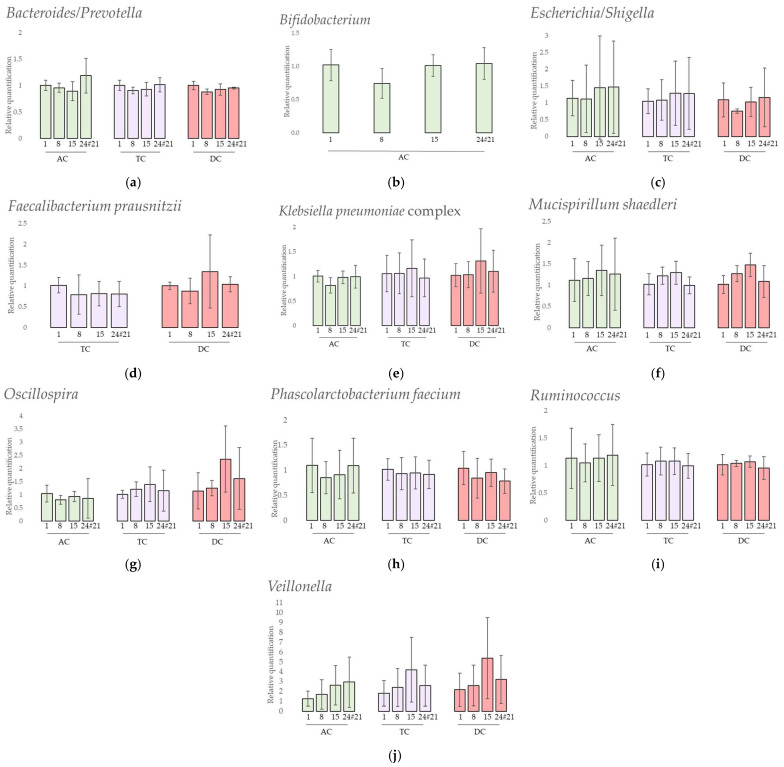
Relative quantification of 10 selected taxa (**a**) *Bacteroides/Prevotella*, (**b**) *Bifidobacterium*, (**c**) *Escherichia/Shigella*, (**d**) *Faecalibacterium prausnitzii*, (**e**) *Klebsiella pneumoniae* complex, (**f**) *Mucispirillum schaedleri*, (**g***) Oscillospira*, (**h**) *Phascolarctobacterium faecium*, (**i**) *Ruminococcus* and (**j**) *Veillonella* assessed by qPCR analysis after one daily ULIP33 phage injection (10 mL at 10^9^ PFU/mL) in the ascending colon for 7 days. Mean fold-changes (±SD) are presented and calculated using 2^−ΔΔCq^ method. AC: ascending colon; TC: transverse colon; DC: descending colon.

The results of the 11 taxa followed by qPCR are presented in Figure 8.

Firstly, the results showed that 1 targeted specie was not detected at all during the experiments: *Akkermansia muciniphila*. Secondly, 1 targeted specie, *Faecalibacterium prausnitzii*, was only detected in TC and DC samples (Figure 8d). Thirdly, *Bifidobacterium* was not detected in all samples of the experiment. In AC (Figure 8b), it was not detected at day 1 and day 15 for repetition 1 and at day 8 for repetition 2. In TC and DC, it was only detected at the end of the experiment in repetitions 2 and 3.

A statistical analysis was performed to highlight relative quantification changes following treatment. Either ANOVA tests or Friedman tests, depending on the normality and homoscedasticity of the distributions, were applied. In AC, *Bacteroides*/*Prevotella*, *Escherichia*/*Shigella*, *Oscillospira*, *Phascolarctobacterium faecium,* and *Ruminococcus* results presented non-parametric distributions while *Klebsiella pneumoniae* complex, *Mucispirillum schaedleri*, *Bifidobacterium,* and *Veillonella* showed parametric distributions. In TC, *Escherichia*/*Shigella*, *Klebsiella pneumoniae* complex, *Mucispirillum schaedleri*, *Oscillospira*, *Phascolarctobacterium faecium* and *Ruminococcus* presented non-parametric distributions while the other selected taxa showed parametric distribution. In DC, only *Klebsiella pneumoniae* complex and *Ruminococcus* presented parametric distributions. Independently from the applied statistical test, no significant changes in the relative quantification were observed in any colon and for any selected bacteria.

## 4. Discussion

Phage therapy is a promising approach for fighting antimicrobial resistance. However, proofs of the safety of this antibiotics’ alternatives are needed, especially for the impact on the human intestinal microbiota. Indeed, whether to fight bacterial digestive diseases or to use the digestive tract as the route of medicine administration, the impact of phage on the bacterial ecosystem in the colon is an important factor that must be taken into account. In this context, the SHIME^®^ model was a suitable in vitro model to simulate the in vivo conditions encountered in the ascending (AC), transverse (TC) and descending (DC) colons.

The SHIME^®^ system is a well-known model to highlight the effects of probiotics [57,58,59], prebiotics [60], both [25], or phytochemicals [61] on the gut microbiota. However, the impact of phage therapy on intestinal microbiota has not been investigated in detail using this model and, to the best of our knowledge, only three studies have been published so far. The first presented the effects of repeated injections of a phage cocktail against *Salmonella* Typhimurium in the proximal colon’s microbiota and study the phage’s impact and persistence in this complex microbial community [36]. The second underlined the stable activity of two phages against *K. pneumoniae*, phages 1.2–3 s and MCoc5c, after a short passage (21 h) in either the proximal either the distal colon microbiota [37]. The last investigated the persistence of the phage UZI (isolated against a *Klebsiella aerogenes,* previously named *Enterobacter* aerogenes) in the large intestine microbiota’s ecosystem after one injection (with or without an additional bacterial host injection) [35]. In this third study, a mathematical formula was applied to study the theoretical persistence of the phage into the gastrointestinal model and to check if the phage was mechanically washed out from the system. The mathematical model of our study was based on this last study [35]. Similarly to the phage UZI, phage ULIP33 disappeared faster than expected, if the phage is considered as an inert particle, irrespectively of the colon. However, it stayed detectable for 8 ± 2, 11 ± 1, and 14 ± 2 days after cutting off the treatment in AC, TC, and DC, respectively (versus 9, 17 and 18 days), indicating its persistence in intestinal conditions of the model. Different hypotheses can explain this washout phenomenon. Firstly, the absence of the bacterial host probably prevented the phage replication. Secondly, the experimental conditions could have decreased the lytic activity of ULIP33. Indeed, even if the phage was thermostable until 45 °C and showed a stable lytic activity over a pH between 6 and 10, its lytic activity was decreased after 1 h incubation at pH 4 [38]. Thirdly, a non-host material can interact with the phage and decrease its lytic activity, for example pancreatin or bile salt, as already seen in another study with an *E. coli* phage [62]. Testing the resistance of the phage ULIP33 in the PJ and feed for a long time would be interesting to perform. Fourthly, the automatic acid and base discharges can dilute the phage concentration. Finally, the titration method has also a limit of detection. These assumptions can explain why a lower-than-expected concentration was observed after the treatment in the total volume of the bioreactors.

The production of SCFA, is a key step of bacterial colonization [12]. In healthy conditions, microbioma from fecal samples presents a ratio of acetate, propionate, and butyrate around 60/20/20 [63]. In this study, propionate producer’s species such as *Bacteroides*, *Phascolarctobacterium faecium,* or *Veillonella* [28,29] were well represented and could have led to a higher propionate ratio. In addition, the high proportion of butyrate was probably due to a high proportion of butyrate-producer bacteria such as *Faecalibacterium prausnitzii* or *Roseburia* genus [28,29]. In vivo, SCFA concentration is almost the same in the different part of the colon [64]. In this model, the increase in SCFA concentration from proximal to distal parts of the colon can be explained by the lack of absorption in the model. Regarding the impact of phage addition, no significant SCFA production variation was observed over time after the treatment. This observation is similar to other studies and is promising for phage therapy. In a randomized, double-blind, placebo-controlled crossover intervention trial, a commercial cocktail of *Escherichia coli*-targeting phages was given to healthy volunteers [65]. Feces and blood were sampled to study the impact of the phages on the microbiota and on inflammatory markers, and no impact of the SCFA production was highlighted. In another more recent study, the impact of the phage vB_EcoS_Ace, targeting STEC *E. coli*, on the intestinal microbiota was evaluated in a 24 h in vitro fermentation model [66]. In that study as well, SCFA production showed no significant changes due to the phage addition.

Microbiota profiling is also an important factor for host’ health. In healthy individuals, this complex ecosystem is in a steady state. However, in several situations, this normobiosis can switch to dysbiosis. Microbiota compositions changes are linked to different diseases such as obesity, inflammatory bowel disease, neurological degenerative disease, or diabetes [67,68,69,70,71]. Although phages are an integral part of the gut microbiota [72], phage treatment could disrupt the balance of this biocenosis in the same way as antibiotics [73] or xenobiotics [74].

In general, at the phyla level, 90% of the intestinal microbiota is represented by the phyla Firmicutes and Bacteroidota [75]. In this model, the ratio was closer to 100% in the TC and DC, as observed in the inoculum (data not shown). In the AC colon, the ratio was close to 90% with a higher proportion of enterobacteria (especially *Klebsiella* and *Esherichia-Shigella*), belonging to the Proteobacteria phylum. These differences can be explained by different in vitro conditions in the AC colon compared to the end of the digestive tract, and the fecal inoculum. In this study, with a high representation of the phylum Firmicutes and the family *Lachnospiracae* in the stool sample, the donor’s microbiota was characterized as enterotype 3, the most common enterotype [76,77].

At the genus level, 14 genera were found with a relative abundance higher than 1% in the model: *Anaeroglobus*, *Bacteroides*, *Eisenbergiella*, *Escherichia*-*Shigella*, *Faecalibacterium*, *Klebsiella*, *Lachnoclostridium*, *Lachnospiraceae*_*ge*, *Parabacteroides*, *Phascolarctobacterium*, *Selenomonadaceae*_*ge*, *Sudoligranulum*, *Tyzzerella,* and *Veillonella*. Only some of them were found in higher proportion in the fecal inoculum: *Bacteroides*, *Faecalibacterium*, *Lachnoclostridium*, *Lachnospiraceae*_*ge*, *Parabacteroides,* and *Subdoligranulum*. The bacterial composition of the TC and DC was more comparable to the fecal inoculum than the AC community. Indeed, the in vitro conditions of these parts are close to the in vivo conditions encountered at the end of the gastro-intestinal tract. Moreover, the TC and DC’s in vitro conditions are close to each other and can sometimes be associated as a distal colon [78].

Biological communities can be characterized by diversity index: α and β [79]. In this study, three indexes—Shannon, Simpson, and Piélou—and 1 estimator—Chao 1- were calculated. These indexes are often calculated in studies about the phage’s impact on the gut microbiota and the most commonly used ones are the Shannon index [65,66,80,81,82,83] and the Chao1 estimator [65,81,83]. No significant results were observed after the statistical analysis performed to highlight any changes in these indexes after the phage treatment. Nevertheless, the Piélou index was higher and the Chao1 lower in the AC colon than in the other colons, probably due to the saccharolytic substrate availability which promotes specific bacterial genus growth such as *Veillonella,* a trend already seen in other SHIME^®^ experiments [22,25,84,85]. Regarding the non-significant statistical results, previous in vivo and in vitro studies concluded the same. The in vivo models were based on a human stool sample analysis that was testing phage cocktails against *E. coli* (called PreforPro) [65,80], on infected mice with a *E. coli* O157:H7 and a phage cocktail against *E. coli*, *Salmonella spp.* and *Listeria monocytogenes* (named F.O.P. for Foodborne Outbreak Pill) [81], or on a peritonitis mouse infection model with *Enterococcus faecalis* and a cocktail of the phages EFDG1 and EFLK1 against *Enterococcus* species [82]. The in vitro model tested the phage vB_EcoS_Ace [66] or the FOP phage cocktail in the case of *Listeria monocytogenes* infection [86]. However, in two rodent model studies, the researchers highlighted an increase in the α-diversity in parallel to a hyper-permeability of the gut barrier, which is a signature of intestinal inflammation [83,87].

Then, to compare the diversity between different samples, the Bray–Curtis dissimilarity was measured and showed a high clustering of all the samples, which was even more important in the same repetition samples. The same strong clustering of the samples with or without the phage treatment was highlighted in in vivo studies on human stool analysis and in a peritonitis mouse infection model with *Enterococcus faecalis* or in in vitro model with *Listeria monocytogenes* targeted by cocktail phages [65,80,82,86]. Finally, to mathematically assess the genetic diversity between the samples over time in the same colon, AMOVA and HOMOVA tests were calculated, based on the Bray–Curtis dissimilarity matrix. This showed no significant results. All these parameters lead to the same conclusion: the phage did not impact the β-diversity of intestinal microbiota.

To further investigate the microbiota composition, semi-quantitative analyses were obtained for six genera and five species using qPCR and the delta–delta Ct method [44].

Some of those species are important health promoters such as *Akkermansia muciniphila* and *Bifidobacterium.* The first degrades the intestinal mucus and produces substrates locally, such as monosaccharides and acetate, used by other intestinal bacteria through a cross-feeding phenomenon. *Akkermansia* also plays a role in the gut barrier, immune-modulation, and obesity [26], but was not detected in this donor. The *Bifidobacterium* genus is present in a higher proportion in babies due to their bifidogenic alimentation through milk [88], and nowadays has an use as probiotic for human health [27]. This genus was found in a higher proportion in AC compared to the other colons. This is probably due to the saccharolytic substrate availability in this part of the in vitro model, which can be used by these bacteria [89]. No significant quantification variations were shown after the phage treatment in AC and some bacteria were found at the end of the experiment in TC and DC.

The propionate-producers *Phascolarctobacterium faecium*, *Veillonella,* and *Bacteroides*/*Prevotella* showed no significant quantification variations after the phage treatment. However, the quantification of *Veillonella* slightly increased over time.

The decrease in the quantification of *Faecalibacterium prausnitzii*, a butyrate producer species, in parallel to an increase in *Escherichia*/*Shigella* is a signature of inflammation and inflammatory bowel diseases [30,31]. In this study, the decrease of *F. prausnitzii* was not observed. However, even if no significant changes were obtained after the treatment, the quantification of *Escherichia/Shigella* varied greatly between the repetitions. In the second repetition especially, the quantification of *E. coli*/*Shigella* was increased but not associated with a decrease of *F. prausnitzii*. No *F. prausnitzii* were found in the AC probably due to its sensitivity to acidic pH and bile salts [90].

*Oscillospira, Ruminococcus,* and *Mucispirillum schaedleri* are three genera related to chronic inflammatory bowel disease [32]; *Oscillospira* is negatively correlated and the others are positively correlated. However, all their quantifications remained stable over time after the phage treatment.

The results obtained after the qPCR quantification of *Klebsiella pneumoniae* complex (including, among others, the *pneumoniae* subspecies) showed that this group remained stable even in the presence of the phage. Additionally, no lytic activity on Petri dishes of the phage ULIP33 was observed against the *Klebsiella pneumoniae* found in the microbiota of the donor (data not shown). These results, closely interlinked with the wash out of the phage and the absence of its replication, are supporting arguments for non-off target replication of the phage ULIP33.

Lastly, even if the parameters studied through this work did not reveal any impact of the phage on the gut microbiota, it is important to underline some limitations and characteristics of this in vitro model. Firstly, the in vitro microbial diversity was less diverse than the inoculum with lower OTU numbers due to a specialization of the microbiota to the in vitro conditions, as already seen in other SHIME experiments [22]. On one hand, this specialization is a feature of the model to distinguish the three parts of the colon. On the other hand, this loss of diversity could be due to the sampling, storage, and culture method of the stool samples. Furthermore, as a classical technical replicate, the three repetitions of the experiment were practiced with the same feces inoculum to avoid differences between repetition as already highlighted [25]. However, the bacterial populations implanted in the system were different from one repetition to another. Therefore, even if the results obtained through this study provide interesting information on the restricted effect of the phage ULIP33 on the gut microbiota using the SHIME dynamic in vitro model, further studies are needed for drawing general conclusions. For example, testing the phage effect on other donors’ microbiotas could give more validation to these results. Another example would be to compare the microbiota parameters of a SHIME experiment without phage injection during the whole period of the experiment. The use of an inert tracer to follow the transit in parallel could also be useful to compare the observed persistence of the phage with the tracer. Additionally, the next important step, would be to test the ability of the ULIP33 phage to interact with its host, the *K. pneumoniae* SB4385, in this same model.

## 5. Conclusions

In this experiment, different parameters were analyzed to highlight the impact of the phage vB_KpnP_K1-ULIP33 on the intestinal microbiota in the SHIME^®^. Specifically, SCFA production, relative bacterial abundance including clustering and ecosystem diversity (α and β), and qPCR targeting specific bacteria of interest were deeply investigated. Even if some variations can be observed, probably due to the model, we demonstrated in this study that the phage did not impact the microbiota inoculated into the SHIME^®^ system.

## Figures and Tables

**Table 1:** Average concentrations (in mM) of acetate, propionate, butyrate, total SCFA, and acetate/propionate/butyrate ratio observed in the ascending, transverse, and descending colons after daily ULIP33 phage injection (10 mL at 10^9^ PFU/mL) in the ascending colon for 7 days.

Colon	Key-Points	Acetate (mM)	Propionate (mM)	Butyrate (mM)	Total (mM)	Ratio (%)
**Ascending**	1	21 ± 3	18 ± 4	11 ± 2	49 ± 5	42/36/22
	8	15 ± 4	18 ± 6	11 ± 2	43 ± 5	34/41/25
	15	16 ± 4	18 ± 2	12 ± 1	46 ± 4	35/39/26
	24#21	13 ± 4	16 ± 4	11 ± 2	39 ± 4	32/40/28
**Transverse**	1	32 ± 7	20 ± 3	19 ± 1	72 ± 8	45/28/27
	8	35 ± 5	26 ± 5	20 ± 3	80 ± 8	43/32/25
	15	31 ± 11	21 ± 3	18 ± 2	70 ± 8	44/30/26
	24#21	28 ± 2	21 ± 2	18 ± 2	67 ± 5	42/31/27
**Descending**	1	37 ± 10	20 ± 4	20 ± 2	77 ± 10	48/26/26
	8	31 ± 12	21 ± 6	17 ± 3	70 ± 9	45/30/25
	15	39 ± 4	27 ± 4	22 ± 2	87 ± 8	44/31/25
	24#21	32 ± 1	22 ± 3	20 ± 4	74 ± 6	43/30/27

Green color: ascending colon; purple color: transverse colon; red color: descending colon.

**Table 2:** The average of the Chao1 estimator, Simpson, Shannon, and Piélou index, and OUT number observed in the ascending, transverse and descending colons after one daily ULIP33 phage injection (10 mL at 10^9^ PFU/mL) in the ascending colon for 7 days.

Colon	Key-Points	Chao1	Simpson	Shannon	Piélou	OTU
**Ascending**	1	31 ± 5.83	0.65 ± 0.02	1.44 ± 0.12	0.39 ± 0.03	28 ± 4
	8	42 ± 4.16	0.70 ± 0.06	1.58 ± 0.22	0.45 ± 0.06	30 ± 3
	15	36 ± 1.54	0.70 ± 0.04	1.60 ± 0.16	0.44 ± 0.04	29 ± 3
	24#21	35 ± 0.58	0.71 ± 0.02	1.64 ± 0.11	0.44 ± 0.03	30 ± 2
**Transverse**	1	79 ± 4	0.71 ± 0.03	1.81 ± 0.18	0.38 ± 0.05	69 ± 2
	8	76 ± 3.91	0.68 ± 0.04	1.67 ± 0.23	0.36 ± 0.06	64 ± 6
	15	78 ± 10.66	0.71 ± 0.04	1.85 ± 0.20	0.38 ± 0.03	68 ± 3
	24#21	82 ± 4.77	0.68 ± 0.05	1.72 ± 0.10	0.32 ± 0.04	68 ± 2
**Descending**	Inoculum	97	0.82	2.58	0.58	83
	1	93 ± 16.13	0.62 ± 0.10	1.55 ± 0.25	0.42 ± 0.04	69 ± 4
	8	83 ± 5.50	0.61 ± 0.09	1.46 ± 0.27	0.40 ± 0.05	70 ± 4
	15	113 ± 38.07	0.66 ± 0.04	1.68 ± 0.17	0.43 ± 0.04	76 ± 5
	24#21	80 ± 11.15	0.57 ± 0.12	1.43 ± 0.17	0.40 ± 0.02	71 ± 6

Green color: ascending colon; purple color: transverse colon; red color: descending colon.

## Data Availability

Not applicable.

## Supplementary Materials

The following supporting information can be downloaded at: https://www.mdpi.com/article/10.3390/v15030719/s1, Table S1: Sequences and annealing temperatures used for each taxon-specific qPCR experiment. Figure S1: Microbial community analysis of fecal sample of the donor assessed by 16S rRNA Illumina Sequencing.
