# Supplementary material for: Impact Assessment of vB_KpnP_K1-ULIP33 Bacteriophage on the Human Gut Microbiota Using a Dynamic In Vitro Model
Source: Viruses. 2023 Mar 10;15(3):719. doi: 10.3390/v15030719 (PMC10057081; doi:10.3390/v15030719)
Supplement: Supplementary file 1 [file viruses-15-00719-s001.zip › viruses-2120547-supplementary.docx]

Impact assessment of vB_KpnP_K1-ULIP33 bacteriophage on the human gut microbiota using a dynamic *in vitro* model

Laforêt F. ^1,2^, Antoine C. ^1,2^, Lebrun S. ^2^, Gonza I. ^2^, Goya-Jorge E. ^2^, Douny C. ^3^, Duprez J.-N. ^1^, Scippo M.-L. ^3^, Taminiau B. ^4^, Daube G. ^4^, Fall A. ^5^, Thiry D. ^1,†^ and Delcenserie V. ^2,*,†^

^1^ Laboratory of Bacteriology, Department of Infectious and Parasitic Diseases, FARAH and Faculty of Veterinary Medicine, University of Liège, 4000 Liège, Belgium; fanny.laforet@uliege.be (F.L.); celine.antoine@uliege.be (C.A.); jean-noel.duprez@uliege.be (J.-N.D.); damien.thiry@uliege.be (D.T.)

^2^ Laboratory of Food Quality Management, Food Science Department, FARAH and Faculty of Veterinary Medicine, University of Liège, 4000 Liège, Belgium; egoya@uliege.be (E.G.-J.); iegonza@uliege.be (I.G.); veronique.delcenserie@ulg.ac.be (V.D.)

^3^ Laboratory of Food Analysis, Department of Food Sciences, FARAH and Faculty of Veterinary Medicine, University of Liège, 4000 Liège, Belgium; cdouny@uliege.be (C.D.)

^4^ Laboratory of Microbiology, Department of Food Sciences, FARAH and Faculty of Veterinary Medicine, University of Liège, 4000 Liège, Belgium; bernard.taminiau@uliege.be (B.T.); georges.daube@uliege.be

^5^ Genalyse Partner SA, En Hayeneux 62, 4040 Herstal, Belgium; afa@genalyse.eu (A.F.)

† These authors contributed equally to this work.

1. Material and Methods

**Table S1**. Sequences and annealing temperatures used for each taxon-specific qPCR experiment

| **N°** | **Target taxon (ID)** | **Primer sequence** | **T°C annealing^1^** | **Reference** |
| --- | --- | --- | --- | --- |
| 1 | Universal bacteria | F: AAACTCAAAKGAATTGACGG  R: CTCACRRCACGAGCTGAC | 61,5 | [44] |
| 2 | *Bacteroides* and *Prevotella* genera | F: GAGAGGAAGGTCCCCCAC  R: CGCTACTTGGCTGGTTCAG | 54 | [47] |
| 3 | *Bifidobacterium* genus | F: GGGTGGTAATGCCGGATG  R: CCACCGTTACACCGGGAA | 54 | [46] |
| 4 | *Akkermansia muciniphila* species | F: CAGCACGTGAAGGTGGGGAC  R: CCTTGCGGTTGGCTTCAGAT | 54 | [45] |
| 5 | *Mucispirillum schaedleri* species | F: CGAGCGTTGTTCGGAGTGACTG  R: CCAGCCAGATTGCCGCCTTC | 51 | [52] |
| 6 | *Ruminococcus* genus | F: GAAAGCGTGGGGAGCAAACAGG  R: GACGACAACCATGCACCACCTG | 50 | [51] |
| 7 | *Klebsiella pneumoniae* species | F: CTAAAACCGCCATGTCCGATTTAA  R: TTCCGAAAATGAGACACTTCAGA | 60 | [53] |
| 8 | *Veillonella* genus | F: AYCAACCTGCCCTTCAGA  R: CGTCCCGATTAACAGAGCTT | 61,5 | [50] |
| 9 | *Phascolarbacterium faecium* species | F: GGCGGCTTAATAAGTCGAGC  R: CGTTCGCTACCCTGGCTTTC | 58 | [49] |
| 10 | *Faecalibacterium prausnitzii* species | F: AGATGGCCTCGCGTCCG  R: CCGAAGACCTTCTTCCTCC | 54 | [47] |
| 11 | *Escherichia* and *Shigella* genera | F: CATGCCGCGTGTATGAAGAA  R: CGGGTAACGTCAATGAGCAAA | 60 | [47] |
| 12 | *Oscillospira* genus | F: AAGGAGTTTTCGGACAACGG  R: ATTCAAGGGGTACCGTCTTC | 55 | [48] |
|  |  |  |  |  |

^1^ Annealing temperatures (Tann),

1. Results

**Figure S1**. Microbial community analysis of fecal sample of the donor assessed by 16S rRNA Illumina Sequencing.

Relative abundance histogram at genus level (in %).

“_ge” : unknown but identified bacteria at genus level and differentiated from the other bacteria of the same taxonomic level
